# Characterization of Carotid Smooth Muscle Cells during Phenotypic Transition

**DOI:** 10.3390/cells7030023

**Published:** 2018-03-18

**Authors:** Haize Goikuria, Maria del Mar Freijo, Reyes Vega Manrique, María Sastre, Elena Elizagaray, Ana Lorenzo, Koen Vandenbroeck, Iraide Alloza

**Affiliations:** 1 Neurogenomiks Neuroscience Department, Faculty of Medicine and Nursing, Basque Country University, 48940 Leioa, Spain; hgoikuria@gmail.com (H.G.); maritxu96@hotmail.com (M.S.); k.vandenbroeck@ikerbasque.org (K.V.); 2 ACHUCARRO Basque Center for Neuroscience, Basque Country University, 48940 Leioa, Spain; 3 Neurology Unit, Basurto University Hospital (BUH), 48013 Bilbao, Spain; marimar.freijoguerrero@osakidetza.eus (M.M.F.); ANAMARIA.LORENZOGARCIA@osakidetza.eus (A.L.); 4 Vascular Surgery and Angiology Unit, BUH, 48013 Bilbao, Spain; mariareyes.vegamanrique@osakidetza.eus; 5 Radiodiagnostic Unit, BUH, 48013 Bilbao, Spain; elizagaray@yahoo.com; 6 IKERBASQUE, Basque Foundation for Science, 48013 Bilbao, Spain

**Keywords:** carotid atherosclerosis, plaque instability, smooth muscle cells, *MYH11*

## Abstract

Vascular smooth muscle cells (VSMCs) are central players in carotid atherosclerosis plaque development. Although the precise mechanisms involved in plaque destabilization are not completely understood, it is known that VSMC proliferation and migration participate in plaque stabilization. In this study, we analyzed expression patterns of genes involved in carotid atherosclerosis development (e.g., transcription factors of regulation of SMC genes) of VSMCs located inside or outside the plaque lesion that may give clues about changes in phenotypic plasticity during atherosclerosis. VSMCs were isolated from 39 carotid plaques extracted from symptomatic and asymptomatic patients by endarterectomy. Specific biomarker expression, related with VSMC phenotype, was analyzed by qPCR, western immunoblot, and confocal microscopy. *MYH11*, *CNN1*, *SRF*, *MKL2,* and *CALD1* were significantly underexpressed in VSMCs from plaques compared with VSMCs from a macroscopically intact (MIT) region, while *SPP1*, *KLF4*, *MAPLC3B*, *CD68,* and *LGALS3* were found significantly upregulated in plaque VSMCs versus MIT VSMCs. The gene expression pattern of arterial VSMCs from a healthy donor treated with 7-ketocholesterol showed high similarity with the expression pattern of carotid plaque VSMCs. Our results indicate that VSMCs isolated from plaque show a typical SMC dedifferentiated phenotype with macrophage-like features compared with VSMCs isolated from a MIT region of the carotid artery. Additionally, *MYH11*, *KLF5,* and *SPP1* expression patterns were found to be associated with symptomatology of human carotid atherosclerosis.

## 1. Introduction

Carotid atherosclerosis is a chronic progressive vascular disease which can lead to symptoms such as stroke [1]. Unstable carotid atheroma plaques are those that can rupture causing an ischemic attack [2,3], however, the precise mechanisms underlying plaque destabilization and consequent rupture remain unclear [4]. Vascular smooth muscle cell (VSMC) dedifferentiation is known to play a role in atherosclerosis development [5]. VSMCs found in healthy arteries represent a mature, fully differentiated stage and express the factors needed to ensure their specific contractile features [6]. These cells also display high plasticity capacity compared with other muscle cell types [7]. In the context of atherosclerosis, VSMCs are believed to dedifferentiate, proliferate, and migrate, thus contributing to plaque formation [5]; however, VSMCs may also display beneficial roles by protecting against plaque rupture through promotion of plaque repair [8,9,10,11]. Therefore, VSMCs play an essential role during plaque development from moderate stage to advanced atheroma plaques [12,13,14]. This process of cellular physiology change is called “switching of VSMCs” and indicates a change in their phenotype from differentiated contractile-state to dedifferentiated synthetic-state, which is considered a pre-requisite for the first stages of plaque development [5]. The characteristics of these two phenotypes, contractile and synthetic, represent the two opposite ends of the spectrum of VSMCs among which VSMCs with intermediate phenotypes are found [15]. In response to distinct signals, VSMCs can switch in either direction, synthetic- or contractile-state; thus, proliferative VSMCs could reacquire some contractile VSMC characteristics when suitable surrounding signals are present [6]. Recently, Shankman et al. [16] reported that the effects of VSMCs during atherosclerosis may be dependent on the phenotypic modulation features. A recent proteomics study disclosed a number of proteins differentially expressed during VSMC phenotypic modulation and, interestingly, most of these are involved in cell migration and adhesion [17]. During phenotypic modulation, VSMCs decrease expression of specific markers of the contractile phenotype and increase those of the synthetic phenotype [6,18]. Thus, changes in contractile proteins such as myosin heavy chain 11 (MYH11) and its relationship to cytoskeletal components have been associated with the phenotype switch of VMSCs [19].

The recognition of the synthetic phenotype mostly relies upon the reduced expression or the absence of cytoskeletal markers of the mature or differentiated phenotype [7,20]. Thereby, the rigorous identification of mature SMCs requires examination of multiple marker genes [6,21]. Although atherosclerosis has been intensively studied in humans, the exact involvement of VSMCs in plaque rupture remains unclear. Current literature tends to be more focused on understanding the role of VSMCs coming from the coronary rather than carotid artery. However, a recent study has demonstrated that the biology of atherosclerosis in these two arterial beds is different, and thus the involvement of VSMCs too. For instance, vulnerable carotid plaques have a thicker fibrous cap and higher prevalence of calcified nodules than coronary plaques, two characteristics in which VSMCs have a role, and that may modulate plaque rupture [22,23]. Due to these differences in the behavior of VSMCs, there is a need of deep knowledge on the role of VSMCs in human carotid atherosclerotic plaques and especially of their involvement in plaque rupture and consequent symptoms. In this study, we have characterized VSMCs from human carotid atheroma plaques through analyzing specific markers for contractile, synthetic, and foam cell/macrophage-like cell phenotypes in two different areas of the plaque; the center of the lesion (PLQ) and the furthest site from the lesion (macroscopically intact tissue, MIT) within the surgically excised carotid area. Moreover, the obtained results of VSMC phenotypes have been correlated with patient symptomatology, with the aim to gain comprehension about the role of VSMCs in plaque destabilization.

## 2. Materials and Methods

### 2.1. Isolation of Human Vascular Smooth Muscle Cells

**Figure 1:**
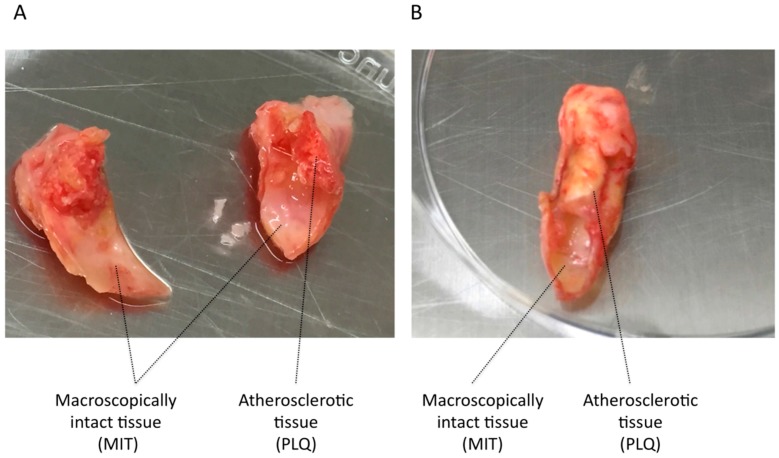
Illustrative photos of carotid endarterectomy specimens. Macroscopically intact tissue and atherosclerotic tissue is visualized on sample from patient 1 (**A**) and patient 2 (**B**).

Carotid endarterectomy (CEA) surgery was carried out at Basurto University Hospital (Bilbao, Spain) in 39 patients of carotid atherosclerosis disease. CEA was performed in symptomatic patients (*n* = 20) who presented a degree of stenosis higher than 70% with previous history of transient ischemic attack or ipsilateral stroke, and in asymptomatic patients (*n* = 19) with a degree of stenosis higher than 80% and no cerebrovascular associated symptoms. All patients underwent an MRI scan and cervical duplex before and after surgery. Demographic and clinical data for these patients are summarized in Table 1. Carotid atheroma plaque samples were placed on ice and processed immediately. VSMCs were extracted from tissue from the plaque site area (PLQ-SMCs) and tissue from the furthest neighboring area from the lesion (MIT-SMCs), for each plaque sample obtained. Figure 1 shows two representative examples of affected and MIT areas used in this study. An enzymatic tissue digestion method was used to isolate and culture VSMCs in two consecutive digestions. First, tissue was digested by 3 h incubation at 5% CO_2_ and 37 °C with 300 U/mL of Collagenase type I (ColI) (17018029, Thermo Fisher Scientific, Waltham, MA, USA) followed by a second digestion over night with 220 U/mL of ColI at 5% CO_2_ and 37 °C. Digested tissue was filtered by a 100 µm nylon Falcon™ Cell Strainer (CLS431752-50EA, Sigma-Aldrich, St. Louis, MO, USA) to remove undigested tissue and subsequently cells were plated in T25 flasks with 5 mL of selective medium, which consists of 231 medium (M231500, Thermo Fisher Scientific, Waltham, MA, USA) that promotes selective VSMC growth, supplemented with 2 ng/mL FGFb (130-093-839, Miltenyi Biotec, Bergisch Gladbach, Germany), 20 ng/mL IGF-1 (130-093-885, Miltenyi Biotec, Bergisch Gladbach, Germany), 0.5 ng/mL EGF (130-0997-749 Miltenyi Biotec, Bergisch Gladbach, Germany), 5 ng/mL Heparin (H3149, Sigma-Aldrich, St. Louis, MO, USA), 5% newborn calf serum (N4762, Sigma-Aldrich, St. Louis, MO, USA), 0.2 µg/mL bovine serum albumin (BSA) (A9418, Sigma-Aldrich, St. Louis, MO, USA), 2 mM L-glutamine (G7513, Sigma-Aldrich, St. Louis, MO, USA), 100 µg/mL Streptomycin, and 100 U/mL Penicillin (P4458, Sigma-Aldrich, St. Louis, MO, USA). Cell density at harvest was 200,000 cells/5 mL in a T25 flask. All experiments were carried out with cells in passage zero in order to keep overall cell characteristics as similar as possible to those in the natural context, and to avoid extended cultivation periods which would influence the expression levels of analyzed genes [14].

This study was approved by the local ethical committee (Ethical committee of clinical research, Basurto University Hospital) and all carotid atheroma plaques were collected from patients who had signed written informed consent. This research was performed in agreement with the principles outlined in the Declaration of Helsinki.

Human iliac arterial smooth muscle cells were obtained from the Coriell Cell Repositories (Coriell AG11545, Camden, NJ, USA). These cells were obtained by explant method of iliac arterial tissue. The culture was grown on flasks coated with 0.1% gelatin (G8090, Sigma-Aldrich, St. Louis, MO, USA) and 199 medium (11150059, Thermo Fisher Scientific, Waltham, MA, USA) supplemented with 0.02 mg/mL ECGS (02-102, Millipore, Billerica, MA, USA) and 0.05 mg/mL heparin (H3149, Sigma-Aldrich, St. Louis, MO, USA), 10% fetal bovine serum (FBS) (F7524, Sigma-Aldrich, St. Louis, MO, USA), 2 mM L-glutamin (G7513, Sigma-Aldrich, St. Louis, MO, USA), 100 µg/mL Streptomycin and 100 U/mL Penicillin (P4458, Sigma-Aldrich, St. Louis, MO, USA) in T25 flasks. Cell density at harvest was 200.000 cells/5 mL in a T25 flask. 

### 2.2. Flow Cytometry

VSMC cultures were trypsinized at cell density of 200,000 cells/5 mL in T25 flasks and then fixed with 4% paraformaldehyde during 10 min at room temperature. VSMCs were permeabilized with 0.5% saponin (S7900, Sigma-Aldrich, St. Louis, MO, USA) in phosphate-buffered saline (PBS) for 5 min and blocked with 1% BSA (A6003, Sigma-Aldrich, St. Louis, MO, USA) for 30 min. Primary antibodies used were anti-alpha smooth muscle actin at 1/100 (ab66133, Abcam, Cambridge, UK), transgelin/SM22 monoclonal antibody at 1/100 (60213-1-Ig, ProteinTech, Manchester, UK) and anti-CD31 (also called platelet and endothelial cell adhesion molecule 1, PECAM1) [EPR3094] antibody at 1/50 (ab76533, Abcam, Cambridge, UK) during 1 h in shaking at room temperature. VSMCs were washed with 5 mM EDTA (E7889, Sigma-Aldrich, St. Louis, MO, USA) and 0.5% BSA in PBS and were incubated with secondary antibodies Alexa Fluor^®^ 488 goat anti-mouse IgG (H + L) (A-11001, Invitrogen, Thermofisher, Carlsbad, CA, USA) and Alex Fluor^®^ 647 goat anti-rabbit IgG (H + L) (A-21244, Invitrogen, Thermofisher, Carlsbad, CA, USA) at 1/500 for 30 min in shaking and darkness. After washing, cells were resuspended in 5 mM EDTA and 0.5% BSA in PBS.

### 2.3. Gene Expression Analysis

RNA was extracted from VSMCs with PureLink^®^ RNA mini kit (12183018A, Thermo Fisher Scientific, Waltham, MA, USA) followed by DNaseI treatment (12185010, Thermo Fisher Scientific, Waltham, MA, USA). RNA was retrotranscribed with High-Capacity cDNA Reverse Transcription Kit (4368814, Applied Biosystems™, Thermo Fisher Scientific, Waltham, MA, USA) using 160 ng of RNA. Gene expression analysis was carried out using Fast SYBR^®^ Green Master Mix (4385614, Thermo Fisher Scientific, Waltham, MA, USA) in ABI7500Fast Real-Time PCR instrument (Applied Biosystems™, Carlsbad, CA, USA). Primers for genes related with contractile phenotype were α-smooth muscle actin (*ACTA2*), *MYH11*, h-caldesmon (*CALD1*), calponin 1 (*CNN1*), and transgelin (*TAGLN*); for synthetic phenotype were myosin heavy chain 10 (*MYH10*), intercellular adhesion molecule 1 (*ICAM1*), secreted phosphoprotein 1 (*SPP1*, also osteopontin), matrix metallopeptidase (*MMP*) 3, 7, 9, tissue inhibitor of metallopeptidase 1 (*TIMP1*), and microtubule associated protein 1 light chain 3 beta (*MAP1LC3B*); primers for transcription factors related with specific SMCs differentiation were MKL1/Myocardin like 2 (*MKL2*), serum response factor (*SRF*), kruppel like factor (*KLF*) *4* and *5*; primers for genes related with macrophage-like phenotype were CD68 molecule (*CD68*) and galectin 3 (*LGALS3*) (Table S1). Glyceraldehyde-3-phosphate dehydrogenase (*GAPDH*), ribosomal protein 41 (*RPL41*) and β-actin (ACTB) were tested as housekeeping genes. The geometric mean of *GAPDH* and *RPL41* was used for data analysis due to their stable gene expression values across samples [24]. PCR amplification efficiencies were in all cases close to 100%. Results were analyzed using ΔCt method. We analyzed gene expression by taking into account the localization of VSMCs, PLQ-VSMCs (*n* = 39), or MIT-VSMCs (*n* = 39) using the Wilcoxon matched-pairs signed rank test. The Mann–Whitney U test was used to analyze gene expression patterns between plaque VSMCs from asymptomatic (*n* = 20) and symptomatic (*n* = 19) patients, as well as the expression levels of 15 μM 7-ketocholesterol-treated HIASMCs versus not-treated HIASMCs (3 independent experiments). Statistical analysis was performed with GraphPad Prism 5 software. *p* value < 0.05 was considered significant.

### 2.4. Western Blot

Protein extraction was carried out with RIPA lysis buffer (150 mM TrisHCL, 150 mM NaCl, 0.5% Deoxycholate, 0.1% SDS, 1% NP-40) for 30 min at 4 °C followed by centrifugation at 20,000× *g* for 10 min in trypsinized VSMCs. Cell lysates were quantified by Pierce™ BCA Protein Assay Kit (23225, Thermo Fisher Scientific, Waltham, MA, USA) and 12 µg protein were loaded into 6% or 12% SDS-PAGE gels with reducing loading buffer. Then, proteins were transferred from SDS-PAGE gel to 0.45 µm-pore PVDF membrane. PVDF membranes were blocked with 2% casein solution following incubation with primary antibody anti-smooth muscle Myosin heavy chain 11 (ab82541, Abcam, Cambridge, UK) and then washed with TBS-Tween. The secondary antibody was anti-rabbit horseradish peroxidase conjugate (#7074, Cell Signaling Technology, MA, USA) and as housekeeping anti-GAPDH antibody (MAB374, Millipore, Billerica, MA, USA) was used for normalization. Proteins were detected with Immobilon™ Western Chemiluminescence HRP Substrate detection reagent (WBKLS0500, Millipore, Billerica, MA, USA) and were visualized with the ChemiDoc™ XRS Imaging System (Bio-Rad, Hercules, CA, USA). Data analysis was done by densitometry comparing MIT (*n* = 7) and PLQ (*n* = 7) cells and additionally, between A (*n* = 6) and S (*n* = 5) plaque VSMCs, both using Image Lab™ Software (Bio-Rad, Hercules, CA, USA).

### 2.5. Confocal Microscopy

Cells were grown on round 14 mm glass coverslips (Thermo Scientific, Waltham, MA, USA), fixed in ice-cold methanol for 10 min at room temperature and then blocked in PBS/3% *w*/*v* BSA (A6003, Sigma-Aldrich, St. Louis, MO, USA) for 30 min and stained with primary antibody anti-smooth muscle Myosin heavy chain 11 (ab82541, Abcam, Cambridge, UK) for 1 h at room temperature. Cells were washed in PBS pH 7.45 and incubated with Alexa Fluor^®^ 488 goat anti-rabbit IgG (H + L) (A-11008, Invitrogen, Thermofisher, Carlsbad, CA, USA) secondary antibody for 45 min at room temperature in darkness and DNA was counterstained with 4′,6-diamino-2-fenilindol (DAPI). After three washes, coverslips were mounted in Fluoromount™ Aqueous Mounting Medium (F4680, Sigma-Aldrich, St. Louis, MO, USA). Image acquisition was performed with Leica TCS STED CW SP8 Super-Resolution Microscope with a 40× lens and recording optical sections every 0.3 µm. Image analysis was done analyzing MYH11 staining fluorescence of each cell per cell area in MIT and PLQ VSMCs with ImageJ software (National Institute of Health, Bethesda, MD, USA).

## 3. Results

### 3.1. VSMC Isolation from Human Carotid Atheroma Plaque

**Figure 2:**
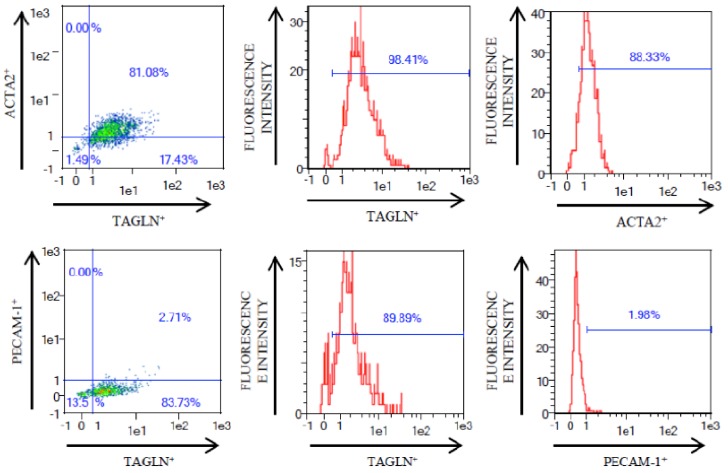
Illustrative graphs of flow cytometry of VSMCs isolated from human atherosclerotic carotid artery. ACTA2 and TAGLN were positive markers of VSMCs while PECAM-1 was negative for these cells.

Human carotid atherosclerotic plaque tissues obtained by carotid endarterectomy surgery were enzymatically digested and once cells were totally disaggregated from tissue, we routinely analyzed the presence of two specific markers of VSMCs i.e., ACTA2 and TAGLN, together with that of endothelial cell marker *PECAM1* by flow cytometry in 39 PLQ-VSMCs and 39 MIT-VSMCs. VSMC suspension was 90% ACTA2^+^ and almost 100% of VSMCs isolated from carotid tissue were TAGLN^+^, whereas endothelial cell marker PECAM-1 was nearly absent with just 2% of cells expressing it, together indicative for VSMCs cultures devoid of endothelial cells following cultivation in VSMC-selective culture medium. Figure 2 shows an illustration of flow cytometry detection of those markers in one representative cell culture. 

### 3.2. VSMC-Specific Gene Expression Pattern Shows Phenotypic Modulation in VSMCs Isolated from Human Carotid Plaques

**Figure 3:**
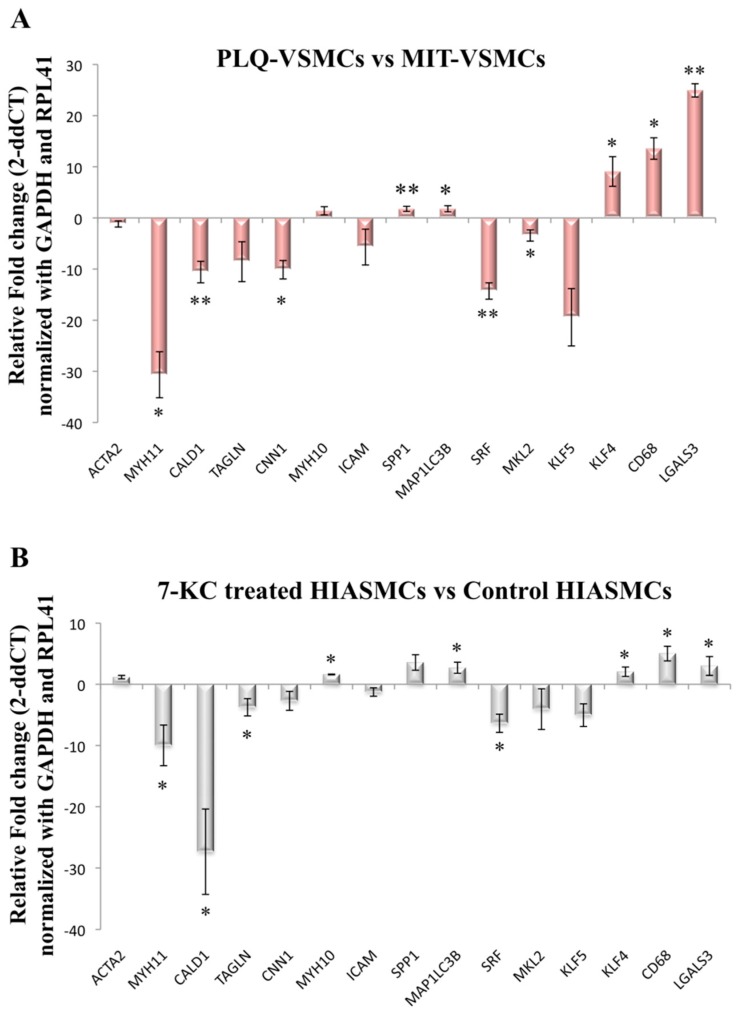
(**A**) Fold change differences of contractile and synthetic marker expression in 39 plaque VSMCs (PLQ-VSMCs) versus 39 macroscopically intact tissue area VSMCs (MIT-VSMCs) analyzed by quantitative PCR (error bars represent ± SEM *n* = 39). Data was normalized with GAPDH and RPL41. Wilcoxon matched-pairs signed rank test (*p* < 0.05 * and *p* < 0.01 **); (**B**) Fold change differences of contractile and synthetic marker expression in 7-ketocholesterol treated (15 μM) HIASMCs versus not treated human iliac arterial SMCs (HIASMCs) analyzed by quantitative PCR (error bars represent ± SEM *n* = 3). Non-parametric Mann–Whitney U test was used (*p* < 0.05 *).

Gene expression levels of contractile and synthetic phenotype markers were analyzed in VSMCs isolated from both the lesion and MIT areas of carotid atherosclerosis plaques. Although *ACTA2* expression did not show any differences, *MYH11*, *CALD1,* and *CNN1* appeared to be downregulated in the lesion area compared to the MIT area (Figure 3A). *MYH11* displayed decreased expression levels (*p* = 0.03) in PLQ-VSMCs compared to MIT-VSMCs with a fold change (FC) of −30.67, and similarly, *CALD1* (*p* = 0.001) was decreased with an FC value of −10.59. In contrast, *TAGLN, MYH10,* and *ICAM1* expression was similar between PLQ-VSMCs and MIT-VSMCs (Figure 3A). The synthetic indicator *SPP1* was upregulated in PLQ-VSMCs (*p* = 0.009). Furthermore, *MAP1LC3B*, which is related to phenotype switching by playing a role in contractile protein removal, was also significantly overexpressed in PLQ-VSMCs vs. MIT-VSMCs (*p* = 0.0181) (Figure 3A).

Human iliac arterial smooth muscle cells (HIASMCs) from a healthy donor treated with 7-ketocholesterol (7-KC) were used to mimic the physiological environment to which SMCs are exposed during atherosclerosis development. HIASMCs showed also a marked decrease of contractile biomarkers when treated during 24 h with 15 μM 7-ketocholesterol (Figure 3B), an oxysterol which is abundant in the plaque.

Transcriptional regulatory pathways control the expression of VSMCs markers and are governed by the transcription factors *SRF*, *MKL2*, *KLF4*, and *KLF5*. *SRF* and *MKL2* were both downregulated in PLQ-VSMCs (Figure 3A). *KLF4*, which has been reported to suppress expression of SMCs markers, at least in part by disrupting SRF–myocardin interaction, showed higher levels of expression in PLQ-VSMCs [16]. On the other hand, *KLF5* expression did not differ significantly between PLQ-VSMCs and MIT-VSMCS (Figure 3A). HIAVSMCs treated with 7-KC showed a similar pattern of transcription factor expression levels as VSMC isolated from carotid plaques (Figure 3B) with SRF diminished and KLF4 enhanced upon 7-KC treatment.

In a recent study, VSMCs isolated from carotid atherosclerotic plaques were found to express a series of macrophage markers [14], concomitant with a macrophage-like phenotype [25]. Analysis of the expression levels of *CD68* and *LGALS3* showed that both macrophage markers were significantly augmented in PLQ compared to MIT-VSMCs (Figure 3A). Similarly, when HIASMCs were treated with 7KC, both genes were clearly upregulated (Figure 3B). Thus, VSMCs in the lesion have been driven towards a phenotype with partial characteristics of macrophages. 

To verify that these differences were not due to symptomatology, we analyzed the gene expression data separately using either only symptomatic samples (20 PLQ-VSMCS vs. 20 MIT-VSMCs) (Figure S1A) or only asymptomatic samples (19 PLQ-VSMCS vs. 19 MIT-VSMCs) (Figure S1B). As similar patterns of expression were observed in both cases, the differences in gene expression found (Figure 3A) are independent of symptomatology and due to the location within the excised carotid area.

### 3.3. MYH1, SPP1, and KLF5 Emerge as Differentially Expressed Genes between Symptomatic and Asymptomatic Carotid VSMC

The gene expression pattern in atheroma PLQ-VSMCs isolated from symptomatic and asymptomatic patients was also analyzed. We found *MYH11* and *KLF5* to be significantly underexpressed in VSMCs from symptomatic patients compared with those from asymptomatic patients (*p* = 0.045 and *p* = 0.01 respectively, Table 2), while *SPP1* was upregulated (*p* = 0.05). 

### 3.4. MYH11 Expression in VSMCs from Symptomatic vs. Asymptomatic Patients and from PLQ vs. MIT Region

**Figure 4:**
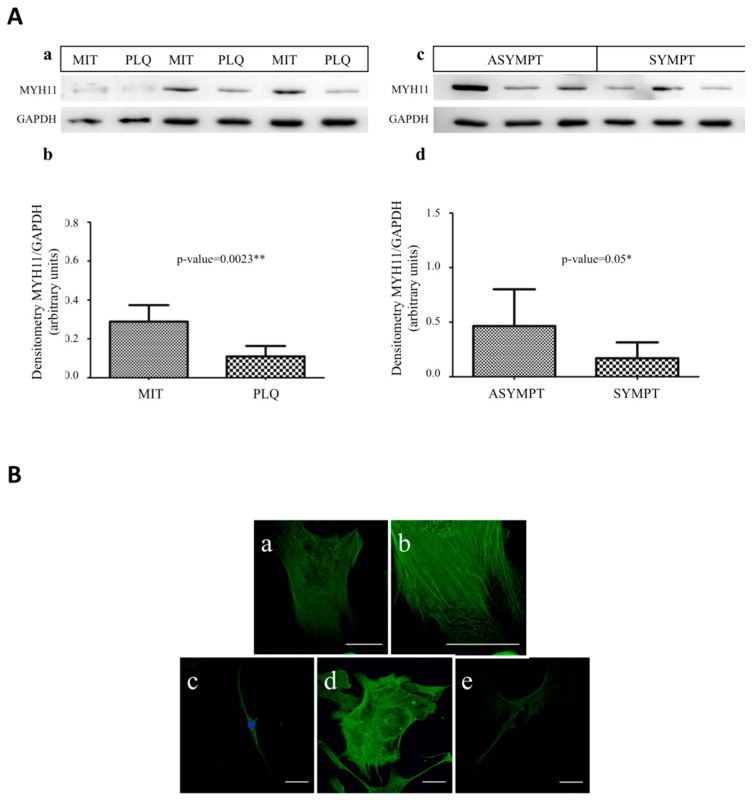
(**A**) MYH11 protein expression in carotid atherosclerotic VSMCs. (**a**,**b**) Western Blot of MYH11 protein in plaque or adjacent MIT area VSMCs, and asymptomatic or symptomatic plaque VSMCs, respectively; (**c**,**d**) graphic representation of A and B show the average of densitometry values of the bands respect to GAPDH protein expression. (MIT: macroscopically intact tissue area VSMCs; PLQ: plaque VSMCs; ASYMPT: VSMCs from asymptomatic plaques; SYMPT: VSMCs from symptomatic plaques) (*p* < 0.05 * and *p* < 0.01 ** were considered statistically significant); (**B**) Immunofluorescence of carotid atherosclerotic VSMCs with MYH11 and nuclei staining. (**a**) MIT-VSMC myofilaments extend throughout the cell forming a network with weaker MYH11 staining in the cell periphery; (**b**) with 2.00 zoom focusing on the cell periphery where MYH11 staining appears as spots; (**c**,**d**) show MYH11 staining in MIT-VSMCs (c image shows elongated and spindle-shaped cell; and d, flattened and polarized cell with lamellipodia); (**e**) PLQ-VSMCs present low fluorescence intensity of MYH11 protein and appear forming thin myofilaments. Scale bar 50 μm.

In immunoblot analysis, MYH11 levels were underexpressed both in PLQ-VSMCs compared with MIT-VSMCs (Figure 4A, panels a and b) and in VSMCs coming from symptomatic versus asymptomatic patients (Figure 4A, panels c and d). 

Immunocytofluorescence analysis with anti-MYH11 by confocal microscopy was performed in carotid VSMCs with the aim to describe the contractile system. PLQ-VSMCs and MIT-VSMCs were labeled with anti-MYH11 and DAPI to identify nuclei. Individual myofilaments were not found to be parallel to the long axis of the cell but formed a network of filaments, and they appeared distributed in the cytoplasm in all single planes of the Z-stacks images, from the base (at the cover slip) to the top of the cell. Z-stack images showed homogeneous distribution of myosin filaments creating a thick fiber-mesh in the base of the cell; myosin-containing stress fibers extended as a network towards the periphery of the cell (Figure 4B, panel a), whereas fibers usually end in extending lamellae, and MYH11 appeared as continuous spots (Figure 4B, panel b) [26]. In the upper planes of Z-stacks there was predominant staining of MYH11 in the central region of the cytoplasm with weaker staining in the cell periphery, which could be observed in MIT-VSMCs (Figure S2, upper panels), whereas there was lack of this pattern in PLQ-VSMCs cells (Figure S2 lower panels). Additionally, in this study, we found that MIT cells showed a higher volume of myofilaments per cell area (Figure 4B, panels c and d) than those VSMCs from the center of atheroma plaque (Figure 4B, panel e), which exhibit less fluorescence intensity of myosin staining.

### 3.5. MMP3, MMP7, MMP9, and TIMP1 Do Not Emerge as Differentially Expressed in VSMCs Coming from Asymptomatic and Symptomatic Patients or between PLQ and MIT Area

The balance between the matrix accumulation and degradation determines plaque stability. We analyzed metalloproteinase 3, 7, 9 and metalloproteinase inhibitor 1 expression levels in our VSMC samples (symptomatic/asymptomatic and PLQ/MIT) (Figure S3A,B). No significant association was found between expression levels of *MMP3*, *MMP9*, *MMP7,* and *TIMP1* and phenotype of VSMCs (symptomatic vs. asymptomatic samples, or PLQ vs. MIT regions).

## 4. Discussion

This study has documented the expression patterns of relevant genes associated with phenotypic modulation in VSMCs by comparing carotid atherosclerotic lesions with macroscopically intact regions located within the surgically excised carotid artery areas. In addition, the VSMCs’ expression pattern was compared between those from symptomatic versus asymptomatic to evaluate if any changes were associated with symptomatology or plaque rupture.

VSCMs express MKL2, which is a transcriptional co-factor for SMC-specific genes [27]. Myocardin binds to the globally expressed transcription factor SRF, and this complex regulates the expression of SMC-specific markers. SMC contractile specific genes contain a functional CArG box in their promoter region, to which the myocardin–SRF complex binds in order to promote SMC gene expression. The expression of transcription factors SRF and MKL2 was decreased in PLQ-VSMCs compared to MIT-VSMCs. This result suggests that cells present in damaged areas have gone through a phenotypic modulation possibly regulated by the SRF transcriptional pathway [16].

Furthermore, KLF-4 is a member of a large family of zinc finger-containing DNA-binding transcription factors and is known to regulate the expression of SMC markers [28] since it is a repressor of SRF/myocardin [29]. Krüppel-like factors have been identified as atheroprotective factors [30] and KFL4, specifically, has been identified as a critical marker that regulates SMC phenotypic transition inhibiting expression of contractile genes. Contrary to this, it has also been suggested to play a pro-atherogenic role due to its involvement in inhibiting the cell proliferation cycle. *KFL4* expression is very low under basal conditions; however, injury promotes its expression in vascular tissues [31]. This evidence supports our observation that KLF-4 is upregulated in PLQ-VSMCs, suggesting that de-differentiated VSMCs are mainly restricted to the area of the lesion, while MIT-VSMCs seem to remain predominantly contractile.

VSMCs keep their plasticity and can undergo cellular modulation from contractile to synthetic-state or reverse the phenotype toward the contractile stage in response to changes in local environmental cues [32]. Upon injury in atherosclerosis, when remodeling is required, VSMCs are able to switch between distinct phenotypes with a trend towards increased rates of proliferation and migration accompanied by enhanced extracellular matrix component production. Also, calcification or release of inflammatory signals is coordinated with a decrease in VSMC contractile markers [6]. The contractile function of VMSCs is achieved by expression of a unique repertoire of proteins such as ACTA2, MYH11, CALD1, and SM22α/TAGLN [7]. We demonstrate here that carotid PLQ-VSMCs showed a decreased expression of specific contractile phenotype markers MYH11 (protein and mRNA level) and CALD1 (mRNA level) compared to MIT-VSMCs. MYH11 has been identified as a marker for specific SMC lineage [6,33] and is a late marker during development, expressed only in fully differentiated cells and showing a high degree of specificity for contractile phenotype VSMCs [34]. In addition, data obtained from HIASMCs from a healthy donor treated with 7KC, one of the major components of oxidized lipoproteins in atheroma plaques, indicates that healthy VSMCs under these conditions proceed towards a de-differentiation process. Furthermore, decreased levels of VSMC contractile markers have been reported before in atherosclerosis [6]. Thus, our results suggest that PLQ-VSMCs might have a decreased contractile capacity.

The process of VSMC phenotypic modulation is associated not only with changes in expression levels of contractile proteins but also with the reorganization of these proteins within the cells [9]. In our study, we found VSMCs from PLQ and MIT to be positive for MYH11 protein staining by immunofluorescence microscopy and that PLQ-VSMCs showed a lower volume of myofilaments per cell area with lower fluorescence intensity of the protein, in line with the results of a previous work in which significantly reduced MYH11 protein staining was observed in switched VSMCs coming from atherosclerotic carotids [19]. In addition, Song and coworkers [35] suggested that myofilament disassembly, especially in the upper cortex, could reflect SMC phenotypic modulation, contractile components degradation, and reorganization of contractile and cytoskeletal proteins within the cell [36]. Similarly, our analysis performed by z-stack to observe the whole thickness of cells showed that staining of MYH11 was predominant in the central region of the cytoplasm in the upper planes of the MIT-VSMCs, unlike PLQ-VSMCs that displayed less prominent myosin staining in the perinuclear area, in which myofilaments appeared to be disassembled, such Saltis et al. [37] reported before. Therefore, immunofluorescence microscopy shows that MYH11 in MIT-VSMCs forms strong myofilament bundles, whereas they appear as thin and disassembled fibers in PLQ-SMCs, which is characteristic of phenotype modulation in VSMCs.

We have also found increased levels of *CD68*, *LGALS3*, and *SPP1,* together with the reduced expression of contractile proteins in PLQ-VSMCs compared to VSMCs from MIT area, suggesting that PLQ-VSMCs may have undergone a phenotypic alteration towards a macrophage-like cell to adapt to such changes. This is consistent with results reported before [38,39], demonstrating that VSMCs have the potential to become macrophage-like cells with a capacity to migrate and to display phagocytic behavior to digest excessive material present at the lesion site. In line with this notion, we have also analyzed the expression of the autophagy marker *MAP1LC3B*, which is known to be important in the de-differentiation of VSMCs through removing contractile proteins by autophagy [40] and in carotid plaque stability [41]. Interestingly, PLQ-VSMCs showed higher expression of *MAP1LC3B* than MIT-VSMCs. HIASMCs treated with 7KC also exhibited increased autophagy marker *MAP1LC3B* expression alongside augmentation of *CD68* and *LGALS3*. Thus, similarly to what was reported for mouse SMCs and human coronary SMCs [42], in our study, HIASMCs loaded with cholesterol may initiate conversion towards foam-type cells as indicated by their loss of SMC features and their acquisition of macrophage-type characteristics. VSMC-derived macrophage cells not only make a significant contribution to plaque volume and necrotic core formation but also suppress plaque stability and promote plaque instability [43].

In addition, with the aim to explore any potential correlation between VSMC phenotypes and patient symptomatology, we have analyzed these contractile markers in VSMCs from symptomatic and asymptomatic plaques. Interestingly, plaques showed a similar expression pattern of PLQ vs. MIT for MYH11 expression, i.e., reduced expression at mRNA and protein level in VSMCs coming from symptomatic plaques compared to asymptomatic plaques. This suggests that VSMCs from symptomatic plaques may have suffered a phenotypic modulation, while VSMCs from asymptomatic plaques retain the contractile capacity associated with MYH11 expression [6]. 

Our study results contribute to a better understanding of how VSMCs affect atheroma plaque stability processes, which may be valuable for future therapeutic approaches in carotid atherosclerosis disease.

## Figures and Tables

**Table 1:** Demographic and clinical data from asymptomatic and symptomatic patients. Statistical analysis was performed with the chi-square test for all parameters except age, for which the non-parametric Mann–Whitney U test was used. *p*-value ≤ 0.05 was considered significant.

Patient Characteristics	Asymptomatic	Symptomatic	*p*-Value
*n*	19	20	
Age	68 ± 9	71 ± 8	ns (0.5)
Sex	14 male/5 female	16 male/4 female	ns (0.6)
	***Risk Factors (%)***		
Contralateral occlusion	66	61	ns (1.0)
Hypertension	66	69	ns (1.0)
Diabetes mellitus	51	8	ns (0.09)
Augmented cholesterol	83	62	ns (0.3)
Cardiopathy	0	23	ns (0.2)
Ischemic cardiopathy	41	46	ns (1.0)
Atrial fibrillation	9	23	ns (0.6)
Intermittent claudication	26	24	ns (1.0)
Tobacco	16	8	ns (0.6)
	***Medications (%)***		
Statins	100	70	ns (0.1)
Anticoagulant	8	23	ns (0.2)

**Table 2:** Relative gene expression analysis between 20 symptomatic (SYMPT) VSMCs and 19 asymptomatic (ASYMPT). Gene expression was normalized with the housekeeping genes *GAPDH* and *RPL41*. Differences between the two groups were analyzed with Mann–Whitney U Test. *p*-value ≤ 0.05 was considered significant. (FC, fold change).

Gene Symbol	FC (Asympt vs. Sympt)	*p* Value
Actin, alpha 2, smooth muscle, aorta (*ACTA2*)	1.3	ns
CD68 molecule (*CD68*)	−1.01	ns
Caldesmon 1 (*CALD1*)	−1.17	ns
Calponin 1 (*CNN1*)	1.14	ns
Galectin 3 (*LGALS3*)	1.05	ns
Intercellular adhesion molecule 1 (*ICAM1*)	−1.30	ns
Kruppel like factor 4( *KLF4*)	1.04	ns
Kruppel like factor 5 (*KLF5*)	−1.89	0.01
Microtubule associated protein 1 light chain 3 beta	−1.09	ns
MKL1/myocardin like 2 (*MKL2*)	−1.17	ns
Myosin heavy chain 10 *(MYH10)*	−1.31	ns
Myosin heavy chain 11 (*MYH11*)	−4.53	0.045
Secreted phosphoprotein 1 (*SPP1*)	2.08	0.05
Serum response factor (*SRF*)	−1.38	ns
Transgelin (*TAGLN*)	−1.01	ns

## Supplementary Materials

Supplementary materials are available on http://www.mdpi.com/2073-4409/7/3/23/s1.
