# Supplementary material for: Characterization of Carotid Smooth Muscle Cells during Phenotypic Transition
Source: Cells. 2018 Mar 18;7(3):23. doi: 10.3390/cells7030023 (PMC5870355; doi:10.3390/cells7030023)
Supplement: Supplementary file 1 [file cells-07-00023-s001.pdf]

SUPPLEMENTARY MATERIAL

Characterization of Carotid Smooth Muscle Cells during Phenotypic Transition

Haize Goikuria [1,2], Maria del Mar Freijo [3], Reyes Vega Manrique [4], María Sastre [1,2], Elena Elizagaray [5], Ana Lorenzo [3], Koen Vandenbroeck [1,2,6] and Iraide Alloza [1,2,6,*]

[1] Neurogenomiks Neuroscience Department, Faculty of Medicine and Nursing, Basque Country University, 48940 Leioa, Spain; hgoikuria@gmail.com (H.G.); maritxu96@hotmail.com (M.S.); k.vandenbroeck@ikerbasque.org (K.V.)
[2] ACHUCARRO Basque Center for Neuroscience, Basque Country University, 48940 Leioa, Spain
[3] Neurology Unit, Basurto University Hospital (BUH), 48013 Bilbao, Spain; marimar.freijoguerrero@osakidetza.eus (M.M.F.); ANAMARIA.LORENZOGARCIA@osakidetza.eus (A.L.)
[4] Vascular Surgery and Angiology Unit, BUH, 48013 Bilbao, Spain; mariareyes.vegamanrique@osakidetza.eus
[5] Radiodiagnostic Unit, BUH, 48013 Bilbao, Spain; elizagaray@yahoo.com
[6] IKERBASQUE, Basque Foundation for Science, 48013 Bilbao, Spain
* Correspondence: iraide.alloza@ehu.eus; Tel.: +34-946-018-292; Fax: +34-946-018-289

Table S1. List of primers used in gene expression analysis for VSMC phenotypic characterization.

Gene Symbol	Gene Name	Accession number	Assay code
ACTA2	α-smooth muscle actin	NM_001613	Hs.PT.56a.2542642
ACTB	Actin β	NM_001101	QT00095431
CALD1	H-caldesmon	NM_033138	QT00997899
CD68	CD68 molecule	NM_001040059	Hs.PT.58.2488447.g
CNN1	calponin 1	NM_001229	Hs.PT.58.38799164
GAPDH	glyceraldehyde-3-phosphate dehydrogenase	NM_002046	Hs.PT.39a.22214836
ICAM1	Intercellular adhesion molecule-1	NM_000201	Hs.PT.56a.4746364
KLF4	Kruppel like factor 4	NM_004235	Hs.PT.58.45542593
KLF5	Kruppel like factor 5	NM_001730	Hs.PT.56a.40282397
LGALS3	Galectin 3	NM_001177388	Hs.PT.58.1435723
MAP1LC3B	Microtubule associated protein 1 light chain 3 beta	NM_022818	QT00055069
MKL2	MKL1/myocardin like 2	NM_002446	Hs.PT.58.19760686
MMP3	Matrix metalloproteinase-3	NM_002422	QT00060025
MMP7	Matrix metalloproteinase-7	NM_002423	QT00001456
MMP9	Matrix metalloproteinase-9	NM_004994	QT00040040
MYH11	Smooth muscle-myosin heavy chain 11	NM_002474	Hs.PT.58.2909933
MYH10	Smooth muscle-myosin heavy chain 10	NM_001256012<br>NM_001256095<br>NM_005964	QT00005117
RPL41	Ribosomal protein L41	NM_001035267	Hs.PT.58.38804367
SPP1	Secreted phosphoprotein 1	NM_001040058	Hs.PT.58.19252426
SRF	Serum response factor	NM_003131	Hs.PT.58.4857809
TAGLN	Smooth muscle protein 22-α	NM_001001522<br>NM_003186	QT01678516
TIMP1	Tissue inhibitor of metalloprotesinases-1	NM_003254	QT00084168

A

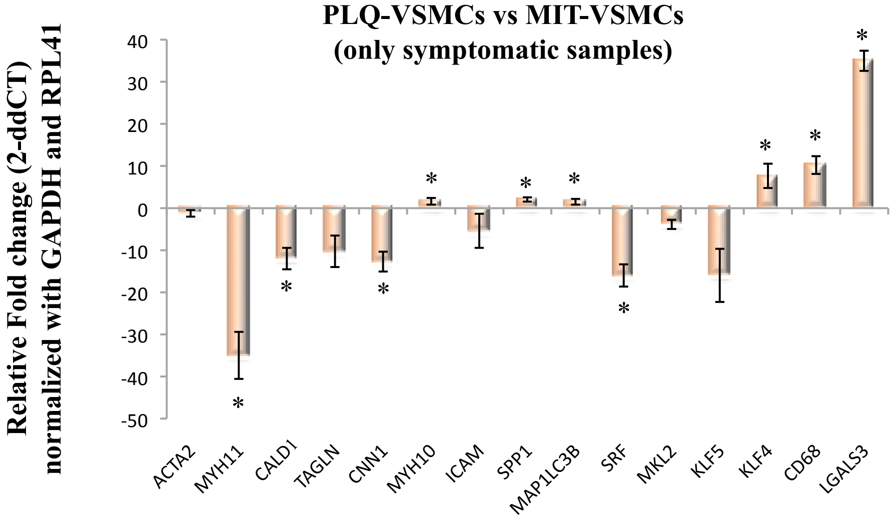

B

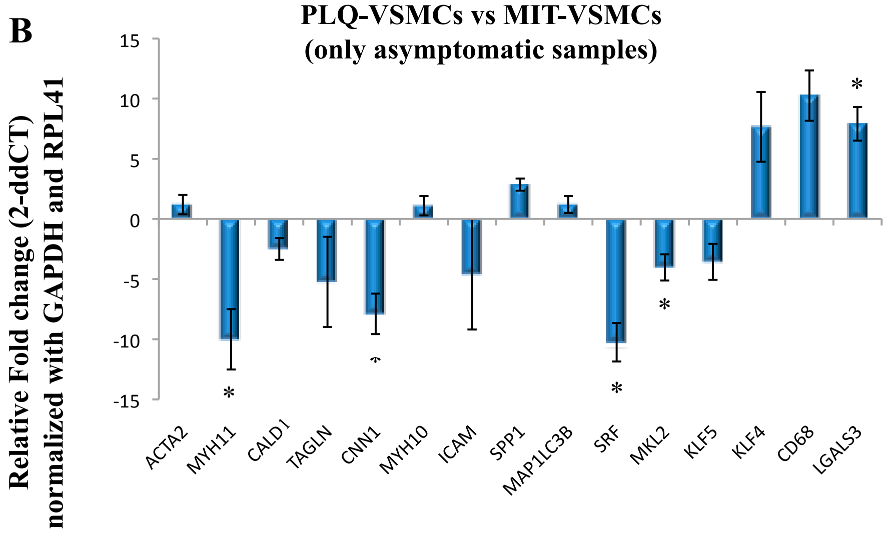

Figure S1. Fold change differences of contractile and synthetic markers in plaque VSMCs (PLQ-VSMCs) versus healthy area VSMCs (MIT-VSMCs) analyzed by quantitative PCR. **(A)** 20 PLQ-VSMCs versus MIT-VSMCs. **(B)** 19 PLQ-VSMCs versus 19 MIT-VSMCs (error bars represent ± SEM, n=20 symptomatic and n=19 asymptomatic). Wilcoxon matched-pairs signed rank test ($p<0.05$*).

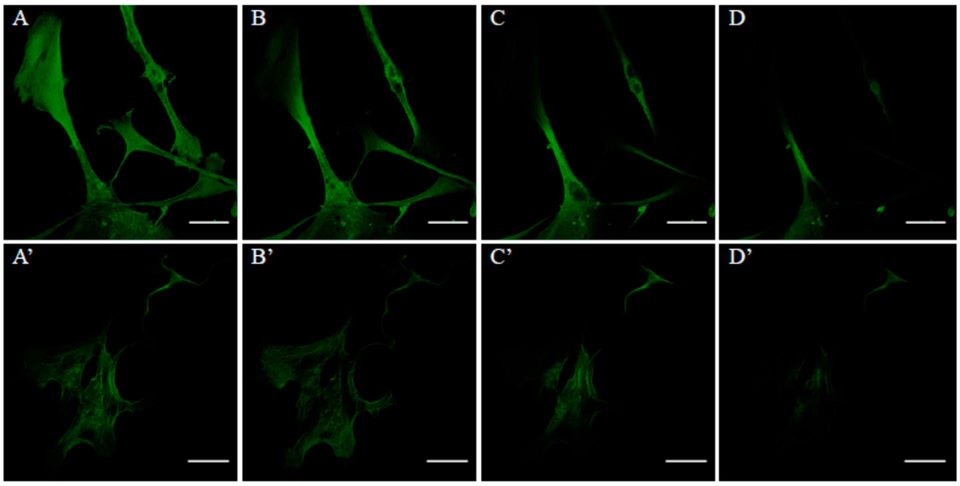

Figure S2. MYH11 staining in VSMCs. **A**, **B**, **C** and **D** VSMCs from adjacent site. 0,9 µm z-stacks from the base o the top of the cells. MYH11 staining appear in perinuclear region in the upper planes of the cell foring thick myofilaments. **A′**, **B′**, **C′** and **D′** VSMCs from atheroma plaque. 0,9 µm z-stacks from the base to the top of the cells, where there is almost lack of MYH11staining and myofilaments appear as thin fibers. Scale bar 50µm.

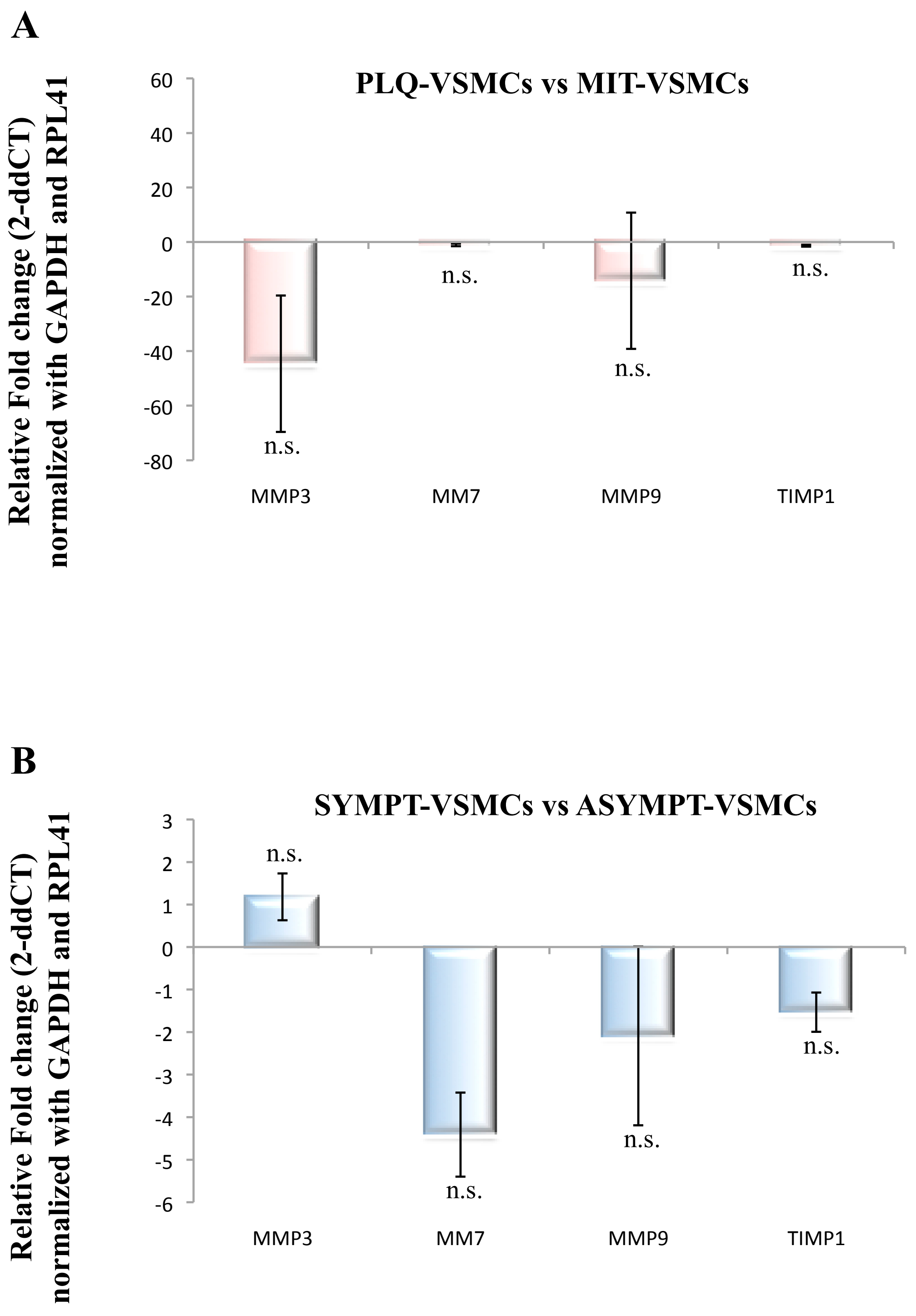

Figure S3. MMMP3, 7, 9, tissue inhibitor MMP 1 mRNA expression quantified by qPCR in plaque VSMCs and adjacent site VSMCs. **(A)** PLQ-VSMCs versus MIT-VSMCs. **(B)** SYMPT-VSMCs versus ASYMPT-VSMCs. Wilcoxon matched-pairs signed rank test was used ($p<0.05$ was considered statistically significant). (n.s. no significant)
